# High correlation between microbubble contrast-enhanced ultrasound, magnetic resonance and histopathology in the evaluation of hepatocellular carcinoma

**DOI:** 10.1590/S1679-45082013000400017

**Published:** 2013

**Authors:** Marcos Roberto Gomes de Queiroz, Miguel José Francisco, Rodrigo Gobbo Garcia, Antonio Rahal, Paolo Salvalaggio, Marcelo Buarque de Gusmão Funari

**Affiliations:** 1 Hospital Israelita Albert Einstein, São Paulo, SP, Brazil

**Keywords:** Carcinoma, hepatocellular/diagnosis, Carcinoma, hepatocellular/pathology, Transplantation, Microbubbles, Magnetic resonance imaging

## Abstract

**Objectives:**

To evaluate the efficacy of microbubble contrast ultrasound in diagnosis of hepatocellular carcinoma and to compare its results with those of magnetic resonance and histopathology.

**Methods:**

A total of 29 patients suffering from chronic liver diseases and awaiting liver transplants at *Hospital Israelita Albert Einstein* were subject to magnetic resonance, microbubble contrast ultrasound, and excision liver biopsies.

**Results:**

Excellent agreement between magnetic resonance and microbubble contrast ultrasound was observed in this study. There was moderate agreement between both imaging methods and histopathology results.

**Conclusion:**

Microbubble contrast ultrasound was as accurate as magnetic resonance to evaluate hepatocellular carcinoma. These results were confirmed by comparing both methods to histopathological diagnosis.

## RESUMO

**Objetivo:**

Avaliar a eficácia da ultrassonografia com contraste de microbolhas no diagnóstico do carcinoma hepatocelular e comparar seus resultados com os de ressonância magnética e anatomia patológica.

**Métodos:**

Foram avaliados 29 hepatopatas crônicos candidatos ao transplante pelo programa de transplante hepático do Hospital Israelita Albert Einstein. Esses pacientes foram submetidos a ressonância magnética, ultrassonografia com contraste de microbolhas e biópsia hepática excisional.

**Resultados:**

Houve concordância ótima entre os resultados da ultrassonografia com contraste de microbolhas e aqueles da ressonância magnética. Notou-se concordância moderada entre os resultados obtidos pelos dois métodos, quando comparados com os resultados de anatomia patológica.

**Conclusão:**

A ultrassonografia com contraste de microbolhas mostrou-se um método tão acurado quanto a ressonância magnética na avaliação do carcinoma hepatocelular, resultado corroborado quando realizada a comparação conjunta dos dois métodos com a anatomia patológica.

## INTRODUCTION

Hepatocellular carcinoma (HCC) ranks third as a cancer-related cause of death worldwide and its incidence increases annually^(1,2)^. Additionally, HCC has been diagnosed more frequently in younger patients, especially in areas where hepatitis B and C viruses are endemic^(1,3)^.

Because of the growing importance of this severe health problem, there is a trend towards enrolling chronic liver disease patients, such as those with alcoholic, viral, or idiopathic causes, with high risk of developing HCC, into surveillance programs^(4)^. Liver transplants have become an essential part of the treatment of patients with chronic liver diseases, and accurate diagnosis of HCC plays an important role in determining transplantation eligibility, according to the criteria established in Milan^(5)^.

Simple and Doppler ultrasonography^(6)^ have been used in the follow-up of hepatic lesions worldwide. However these methods have limitations in the evaluation of focal lesions such as HCC, especially in the case of small masses. Computerized tomography (CT) and magnetic resonance imaging (MRI), both tridimensional imaging modalities employing contrast-enhancement (iodine-based and paramagnetic contrast agents, respectively), are well-known and have been used successfully for diagnosis of suspected lesions and follow-up of HCC in the last two decades^(7,8)^.

In the last decade, MRI has gained special attention, being considered by many authors as the gold standard imaging method for HCC diagnosis and follow-up^(7,9)^. In spite of its diagnostic accuracy, MRI has disadvantages such as high cost, low accessibility and portability, adverse reactions and contraindications to paramagnetic contrast media, absolute or relative contraindications to magnetic field exposure, such as individuals with pacemakers who cannot be subject to such exposure, and it is not recommended for patients who suffer from claustrophobia. These limitations of MRI have prompted the medical community to search for new techniques with similar accuracy but less disadvantages.

Microbubble contrast ultrasound (MCU) techniques were tested and approved for clinical use in over 50 countries^(10–12)^. Because microbubble contrast agents are not nephrotoxic, they can be safely employed in patients with diminished renal function. They are clinically well tolerated and recommended for patients that should not receive iodine-based or paramagnetic contrast agents, used for CT and MRI, respectively^(13–15)^. As to image quality, MCU is similar to CT and contrast-enhanced MRI in many aspects, including its capacity to show the vascular pattern and degree of differentiation of HCC^(16–18)^. Besides being able to show the morphology and vascular patterns of focal hepatic lesions, MCU can be used to detect metastases^(19,20)^, to guide intervention procedures, and to characterize lesions in other organs, such as bowel, pancreas, breast, kidneys, adrenal glands, and prostate^(21)^.

In spite of all abovementioned advantages and its reported applicability in the characterization of focal hepatic lesions, none of the studies published to date have been able to definitely demonstrate the accuracy of MCU when compared to gold standard techniques, such as MRI and histopathology. In these studies, some patients were evaluated by CT and MRI, and others by MCU, with histopathological examination being reserved exclusively for cases considered inconclusive by either imaging method^(22,23)^. The lack of systematic evaluation of the MCU therefore limits the widespread use of the method.

## OBJECTIVE

The goal of this study was to compare the efficacy of microbubble contrast ultrasound in characterizing hepatocellular carcinoma in patients with chronic liver disease, regardless of etiology, with that of gold standard imaging methods, such as magnetic resonance imaging and histopathology.

## METHODS

### Patients

This was a prospective case series study with a convenience sample selected from a group of patients with chronic hepatic diseases (any etiology) who were awaiting liver transplants and were enrolled in the Liver Transplant Program of *Hospital Israelita Albert Einstein* (HIAE), between June 2008 and June 2009. A total of 29 patients selected during a 1-year period met all inclusion criteria and were evaluated with MCU and MRI. Eighteen of the 29 also had histopathology evaluation of their hepatic lesions, the results of which were similarly compared to those obtained by the two imaging methods.

All transplant candidates were initially evaluated via conventional ultrasound for triage of focal hepatic lesions and to rule out thrombosis of the portal vein.

Exclusion criteria for the study are listed in chart 1.

**Chart 1:** Exclusion criteria for the study

**Exclusion criteria for liver transplantation**
Portal vein thrombosis, with or without recanalization
Thrombosis of the hepatic vein and/or of its branches
Heart disease with right-to-left shunt, severe arrhythmia, post-acute myocardial infarction
Severe pulmonary disease
**Known hypersensitivity to components of the microbubble contrast agent**
**Contraindications to magnetic resonance imaging**
Pacemakers
Magnetic metal prosthesis
Hypersensitiveness to paramagnetic contrast agent
Claustrophobia (relative – avoidable with sedation)
Renal patients not undergoing dialysis with creatinine clearance under 60
**Patient refusal to sign the Informed Consent Form**

Patients who did not meet any of the exclusion criteria described in chart 1 but presented with any focal hepatic lesions on conventional ultrasound (without contrast) were then subjected to MRI and MCU. Cirrhotic patients with no nodules visible on conventional ultrasound, but with alpha-fetoprotein levels above 50 were also subjected to MRI and MCU.

### Magnetic resonance imaging: applied methodology

Abdominal MRI examinations were performed according to pre-transplant protocols and consisted of a series without contrast followed by a contrast-enhanced series, and by a diffusion-weighted sequence. A 1.5T, high-field magnet was used for all exams (General Electric, Healthcare).

The MRI sequences and parameters are displayed on chart 2.

**Chart 2:** Magnetic resonance imaging sequences and parameters selected to evaluate the liver nodules

Sequence	2D coronal SSFSE	2D axial FRFSE	2D axial FAT FSE	2D axial SPGR IN/OUT	Timing bolus SPGR	Dynamic 3D axial SPGR FAT	2D coronal SPGR FAT
Image options	Fast, Fc, Zip 512, SS	Fast, TRF, Zip 512, Fc, B, SCIC	Fast, Fc, TRF, Zip 512, SCIC	Fast, Zip 512, SCIC	Fast, Real Time	Fast, Zip2	Fast
PSD name	-	FRFSEopt	FRFSEopt	-	-	-	
TE	80	180	80	-	Minimum	Minimum	Minimum
TR	Minimum	4,900	2,800	190	-	PREP AUTO 12	165
TI FLIP	-	-	-	90	90?	-	60
ETL	-	43	20	-		-	-
VB	62.50	31.25	31.25	62.50	31.25	41.67	31.25
SAT	-	-	FAT	-	Si	FAT	FAT
FOV	40*	34*	34*	34*	31	40*	40*
Espes/Gap	7/1	7/1	7/1	7/1	5/0	4.2/0	7/1
Matrix	256×160	256×192	256×192	256×192	256×128	256×160	256×160
NEX	-	1	1	1	1	1	1
RectFOV	0.90	0.75	0.75	0.75	0.75	0.75	1
Frequency	SI	RL	RL	RL	-	RL	SI
FlowComp	Freq	Freq	Freq	-	-	-	-
Number of slabs	20	24	24	24	-	1slab = 42 loc	20
	2 blocks of 12	2 blocks of 12	-		-	
Time	0:25s	0:49s	0:56s	0:24s	0:24s	0:26s	0:27s

Image interpretation was performed by two staff radiologists of HIAE, with demonstrated experience in abdominal MRI and radiology techniques.

The main aspects analyzed in focal lesions were number, location, dimensions, signal behavior in different weighted scans, post-contrast dynamic enhancement pattern, and restriction of diffusion. Based on image characteristics, each lesion was classified as consistent with HCC or consistent with non-HCC nodule. Regarding the choice of terminology for classification, it is worth mentioning that the caseload was relatively low during the study period, given that patients should present with specific requirements, such as being on the transplant waiting list, present focal hepatic lesions and portal vein patency, complying with ethical norms. Enrollment in the study should be consented and many patients did not agree to participate.

Image interpretation was performed blindly and the radiologists did not have access to clinical or laboratory data of the any of the patients. Previous MRI exams were also not available for comparison.

### Microbubble contrast-enhanced ultrasound: applied methodology

All MCU tests were performed by trained radiologists with demonstrated experience in this specific technique, using the same equipment (Philips IU22, convex transducer 5 MHz), with the same mechanical index (0.8). Microbubble contrast agent (Definity^®^ Bristol; dose 0.1mL/10kg) was given intravenously, followed by 10mL of 0.9% saline by bolus after visualization of the lesion of interest.

The analysis of the contrast dynamic behavior was performed both by static and continuous imaging, emphasizing the following phases:

– arterial phase: starting 15 to 20 seconds post-injection and lasting 10 to 20 seconds;
– portal phase: lasting between 20 and 30 seconds, in which the contrast is visualized predominantly within the portal vein and its branches;
– sinusoidal phase: lasts for 6 to 10 minutes. During this phase the contrast agent percolates the sinusoids and the hepatic capillaries producing a bright and homogeneous echo;
– late phase: when the concentration of microbubbles within the hepatic parenchyma decreases significantly and only a few microbubbles remain in circulation.

The need for a static image during the contrast-enhanced phase of the test allows only for proper visualization of lesions that are close to each other, and not of the entire hepatic parenchyma. Because of this limitation, when more than one lesion was present in different view fields, some priorities were established. Namely, the split-bolus contrast injection technique was used followed by microbubble destruction after the first bolus (high mechanical index), in order to make all of the evaluations of nodular lesions equal.

All MCU studies were recorded on DVD for further review when necessary.

Image interpretation was performed by two experienced radiologists with demonstrated expertise in the MCU image interpretation, and who may or may not have performed the original test.

Image analysis was performed randomly. Among the various aspects analyzed for each focal lesion were size, echogenicity and echotexture on conventional images, enhancement pattern and post-contrast dynamic enhancement, as well as wash-in and wash-out times. Given the abovementioned characteristics, each lesion was classified as consistent with HCC or consistent with non-HCC nodule, in the same ways what was done for MRI.

Similar to MRI, MCU image interpretation was performed blindly with omission of patient identification, clinical data, and other laboratory results. Previous imaging results for the same patient were not made available for comparison.

### Histopathology

Histopathological analysis was performed by excisional liver biopsies. The techniques and processing for pathological study followed the protocol established between the Liver Transplantation team and the Pathology Service of HIAE. Histological examination of the explanted liver is considered the gold standard test, and its results had been compared to those from ultrasound and MRI.

This study was approved by the Research Ethics Committee of the institution under number CAAE 0231.0.028.000–08.

### Statistical analysis

All imaging results were compared and confirmed by histological evaluation, when available, performed via TRU-CUT (core) and/or excisional liver biopsy. Agreement between imaging methods and histopathology was evaluated through the k index (Kappa statistics).

## RESULTS

The results of both imaging modalities for 29 patients were analyzed. In order to evaluate the performance of the MCU method, its results as well as the MRI results of a total of 18 patients were compared to their histopathology diagnosis obtained either by excision biopsy (17) or core biopsy (1). Histopathology was not performed on lesions from the remaining 11 patients because they were excluded from the transplant program, died, or had not yet received transplants by the time the study was finished.

**Figure 1:**
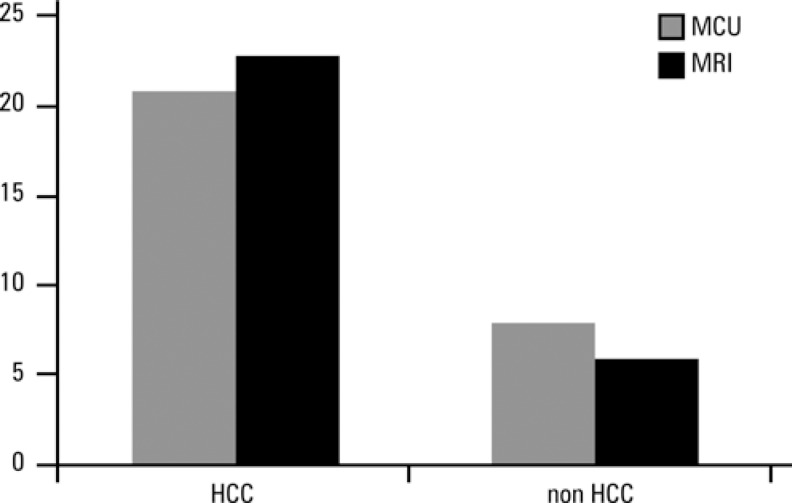
Sensitivity of microbubble contrast-enhanced ultrasound (MCU) and magnetic resonance imaging (MRI) to diagnose hepatocellular carcinoma (HCC) in 29 patients with chronic liver diseases and focal lesions.

Results for MCU and MRI imaging are shown in figure 1. The sensitivity of both imaging methods for detection of HCC in patients with chronic liver diseases was similar.

**Figure 2:**
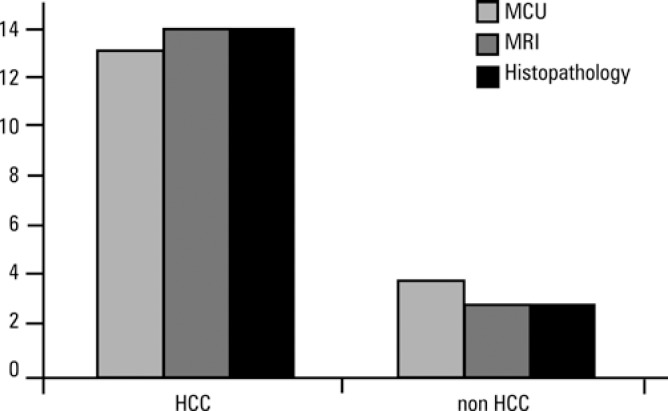
Comparison of microbubble contrast-enhanced ultrasound (MCU) and magnetic resonance imaging (MRI) results and histopathological findings of 18 patients with chronic liver diseases and focal lesions

Like MRI, MCU was an accurate method of detection of HCC in patients with chronic hepatic diseases. Figure 2 shows that MCU and MRI results were similar and comparable to the histopathology diagnosis, considered the gold standard.

To evaluate the agreement between the methods, we used Kappa statistics. The MCU and MRI results were compared to each other (Table 1) and to the histopathological findings (Table 2). All Kappa values obtained were between 0 and 1, with 1 being a perfect agreement.

**Table 1:** Kappa agreement rates for microbubble contrast-enhancement ultrasound (MCU) and magnetic resonance imaging (MRI) for diagnosis of hepatocellular carcinoma (HCC) in patients with chronic liver diseases (n=29)

Non-HCC (MCU)/non-HCC (MRI)	Non-HCC (MCU)/HCC (MRI)
5 (17.2%)	1 (3.4%)
**HCC (MCU)/non-HCC (MRI)**	**HCC (MCU)/HCC (MRI)**
2 (6.9 %)	21 (72.4%)
*Kappa* 0.70 (0.52;0.89)	

**Table 2:** Kappa agreement rates for microbubble contrast-enhancement ultrasound (MCU) and magnetic resonance imaging (MRI) with histopathology for diagnosis of hepatocellular carcinoma (HCC) in patients with chronic liver diseases (n=18)

Test	Non-HCC (test)/non-HCC (H) n (%)	Non-HCC (test)/HCC (H) n (%)
MCU	2 (11.1)	2 (11.1)
MRI	2 (11.1)	1(5.6)
	**HCC (test)/non HCC (H) n (%)**	**HCC (test)/HCC (H) n (%)**
MCU	1(5.6)	13(72.2)
MRI	1(5.6)	14(77.8)
	**Kappa**	
MCU	0.47 (0.16:0.78)	
MRI	0.60 (0.32:0.88)	

H: histopathology

The agreement between the two imaging methods (Table 1) showed a Kappa of 0.70, which is considered good. The 95% confidence interval (95%CI) for this correlation was between 0.52 and 0.89.

Regarding the agreement rates between MCU and HCC, and MRI and HCC (Table 2), there was moderate agreement with a Kappa of 0.47 and 0.6 (MCU and MRI, respectively).

When the three methods were compared, there was a Kappa of 0.63 (95%CI: 0.365–0.899; p<0.001).

## DISCUSSION

Our goal was to evaluate the efficacy of MCU in the detection and characterization of HCC in patients with chronic liver diseases, regardless of etiology. We aimed to validate MCU in light of MRI and H, considered the gold standard diagnostic methods for this disease.

When MCU and MRI imaging methods were compared (29 patients), a good agreement rate was observed. When these imaging methods were compared to histopathology (18 patients), a moderate agreement rate was observed.

Some studies validated MRI as a gold standard imaging method to characterize HCC by showing the excellent agreement rates between MRI and H in the evaluation of focal hepatic lesions^(5,7,9)^.

Taking into consideration that both MCU and MRI had similar agreement rates with histopathology results, one can assume that the two methods are similarly accurate, and in principle, clinically interchangeable. It is important to emphasize that, in our study, the agreement between MRI (and MCU) and H was only moderate. Had enough patients been analyzed, the correlations between MRI and histopathology (and probably between MCU and H as well) would have been expected to be good, as indicated by previous studies. This assumption is supported by the good Kappa value (0.63) obtained when the two imaging methods combined are compared to histopathology.

The international literature reports that more than 3 million MCU exams were performed in Europe until 2010, with no reports of death and extremely rare complications, usually limited to anaphylaxis (1:7000 – 0.014%).

In the literature review we found many studies evaluating the efficiency of ultrasonography and comparing the sensitivity of Doppler ultrasound and MCU^(6,16,18,24)^. However, these studies evaluated extra-hepatic lesions and did not provide information on the applicability of MCU for diagnosis of HCC. Some studies aimed to compare the efficiency of MCU when compared to CT and MRI^(9,15)^, but did not include H. Others still evaluated MCU, MRI, CT, and H, but limited histopathology evaluation in those cases in which imaging was inconclusive^(24–26)^.

The strength of the present study is that it not only compares MCU and MRI, but also compares these two imaging methods with histopathology results on excisional biopsy, which is considered the definitive diagnosis for HCC. This latter comparison renders the results trustworthy. In addition, one of the inclusion criteria for patients in this study was to be awaiting a liver transplant, and therefore compliant with the Milan criteria. Cases that satisfy the Milan criteria, in our opinion, are more homogeneous and less biased. Therefore, the comparison of MCU and MRI imaging, as well as histopathology, within such a homogeneous case population ascribes relevance to the results reported in the present study.

In light of the outcomes of the present study, we suggest that MCU can reliably replace MRI in the characterization and follow-up of HCC in patients with chronic hepatic diseases. It brings imaging features different from those of conventional Doppler ultrasound, since it demonstrates the patterns of vascularization of the nodules at histological perfusion level.

The main limitation of the method is that the liver cannot be evaluated in its entirety and that hypervascular lesions cannot be evaluated simultaneously, rendering this modality inadequate for staging. However, besides being as accurate, MCU is more accessible, more portable, and has lower costs than MRI, the current gold standard modality. Additionally, MCU can be used in cases in which MRI is contraindicated given that microbubble contrast agents are nontoxic and can be used in patients with comorbidities. Future studies with a larger number of patients will be necessary to evaluate the use of MCU in the diagnosis of HCC, supporting our results.

## CONCLUSION

Given the agreement between methods found in this study we suggest that microbubble contrast ultrasound may be as adequate and accurate as magnetic resonance imaging when comparing its findings to histopathology, to characterize and follow-up hepatic nodules, specifically hepatocellular carcinomas, in cirrhotic patients.
